# Cdx1 and c-Myc Foster the Initiation of Transdifferentiation of the Normal Esophageal Squamous Epithelium toward Barrett's Esophagus

**DOI:** 10.1371/journal.pone.0003534

**Published:** 2008-10-27

**Authors:** Douglas B. Stairs, Hiroshi Nakagawa, Andres Klein-Szanto, Shukriyyah D. Mitchell, Debra G. Silberg, John W. Tobias, John P. Lynch, Anil K. Rustgi

**Affiliations:** 1 Division of Gastroenterology, Department of Medicine, Abramson Cancer Center, University of Pennsylvania, Philadelphia, Pennsylvania, United States of America; 2 Division of Gastroenterology, Department of Genetics, Abramson Cancer Center, University of Pennsylvania, Philadelphia, Pennsylvania, United States of America; 3 Penn Center for Bioinformatics, University of Pennsylvania, Philadelphia, Pennsylvania, United States of America; 4 Fox Chase Cancer Center, Philadelphia, Pennsylvania, United States of America; Baylor College of Medicine, United States of America

## Abstract

**Background:**

Barrett's esophagus is a premalignant condition whereby the normal stratified squamous esophageal epithelium undergoes a transdifferentiation program resulting in a simple columnar epithelium reminiscent of the small intestine. These changes are typically associated with the stratified squamous epithelium chronically exposed to acid and bile salts as a result of gastroesophageal reflux disease (GERD). Despite this well-defined epidemiologic association between acid reflux and Barrett's esophagus, the genetic changes that induce this transdifferentiation process in esophageal keratinocytes have remained undefined.

**Methodology/Principal Findings:**

To begin to identify the genetic changes responsible for transdifferentiaiton in Barrett's esophagus, we performed a microarray analysis of normal esophageal, Barrett's esophagus and small intestinal biopsy specimens to identify candidate signaling pathways and transcription factors that may be involved. Through this screen we identified the Cdx1 homeodomain transcription factor and the c-myc pathway as possible candidates. Cdx1 and c-myc were then tested for their ability to induce transdifferentiation in immortalized human esophageal keratinocytes using organotypic culturing methods. Analyses of these cultures reveal that c-myc and cdx1 cooperate to induce mucin production and changes in keratin expression that are observed in the epithelium of Barrett's esophagus.

**Conclusions/Significance:**

These data demonstrate the ability of Cdx1 and c-myc to initiate the earliest stages of transdifferentiation of esophageal keratinocytes toward a cell fate characteristic of Barrett's esophagus.

## Introduction

The normal esophageal squamous epithelium comprises an exquisitely regulated equilibrium between the proliferative basal cell compartment, the differentiating suprabasal cell compartment and the terminally differentiated superficial layer. Migrating cells eventually slough into the lumen due to senescence and apoptosis. A variety of signals trigger epithelial renewal during normal homeostasis and tissue regeneration, the latter due to infections, radiation, and acid/bile exposure. However, prolonged acid exposure, acting alone or in concert with other caustic agents, are believed to cause transdifferentiation that involves the replacement of the normal esophageal epithelium with one that resembles the small intestinal epithelium, designated as incomplete intestinal metaplasia since two of four cell types are evident [1]. These are the columnar enterocytes and the secretory goblet cells, and provide the hallmark features of Barrett's esophagus, in turn a precursor to esophageal adenocarcinoma [2]. While a wealth of epidemiological data exist surrounding Barrett's esophagus and adenocarcinoma [3], [4], the ability to define causative underlying molecular mechanisms, and in particular, the ability to model this condition have remained elusive.

Many genes increase in expression in Barrett's esophagus when compared to the normal epithelium of the esophagus [5]–[10]. Among these genes are the Cdx transcription factors, Cdx1 and Cdx2. These caudal-type homeobox transcription factors determine cell fate in the developing intestine. Cdx1 and Cdx2 induce the primitive epithelium of the intestine and colon toward a glandular cell fate ultimately resulting in the mature intestinal and colonic epithelia of the adult [11]. It is thought that the cell fates of the alimentary canal are determined by the abundance of these transcription factors. Indeed, when Cdx2 is overexpressed in the glandular epithelium of the stomach in mice, intestinal metaplasia occurs [12]–[14]. These data suggest that Cdx transcription factors can shift the cell fates of glandular cells and suggests they may also be involved in the progression of Barrett's esophagus. However, the roles for Cdx in colon cancer and esophageal adenocarcinoma are less clear. Cdx1 gene expression and activity are regulated in colon cancer progression [15]–[17]. Cdx1 expression decreases in adenomatous polyps and correlates with increased dysplasia in Barrett's esophagus. The most recognized mechanism for decreased expression is due to hypermethylation of the Cdx1 promoter of Cdx1 [18], [19]. Interestingly, a similar type of Cdx1 regulation has been observed in Barrett's esophagus and intestinal-type gastric adenocarcinoma [20]. These data suggest that Cdx1 may play a role in the intestinal metaplasia that results in Barrett's esophagus, but may also subsequently serve as a tumor suppressor gene following transdifferentiation.

C-myc is a classic proto-oncogene that is a transcription factor that binds E-boxes as a heterodimer with Max in about 15% of all genes, and recruits co-activators to regulate gene transcription [21]–[24]. C-myc is regulated in part through mitogenic stimuli, and is activated constitutively in cancer cells through gene amplification, chromosomal translocation, point mutation and mitogenic stimulation [25]. The consequences of c-myc transcriptional regulation are protean on cellular behavior, ranging from modulating proliferation, cellular metabolism, apoptosis and differentiation in many diverse cell types [25]. The transcriptional activity of c-myc is dependent upon its ability to dimerize with Max. Mxd1 (Mad1) and Mxi1 (Mad2) are negative regulators of c-myc transcriptional activity and function by binding to Max and sequestering it from c-myc. Regulation of c-myc bound promoter activity is complex and involves several different types of co-activators. They include histone actetlytransferases such as p300/CBP and GCN5; ATPases including TIP48 and TIP49; and the Mediator complex as well as the several others through c-myc's interaction with TRRAP [26]–[30].

Through a microarray comparison of the normal stratified squamous epithelium of the esophagus, Barrett's esophagus and the normal small intestine, and accompanying validation, the molecular signature of Barrett's esophagus highlights the importance of both Cdx1 and c-myc. We find that both transcription factors cooperate to induce the earliest stages in this transdifferentiation program towards Barrett's esophagus as revealed by organotypic culture (3-dimensional or 3D culture) that mimics the tissue microenvironment.

## Results

**Figure 1:**
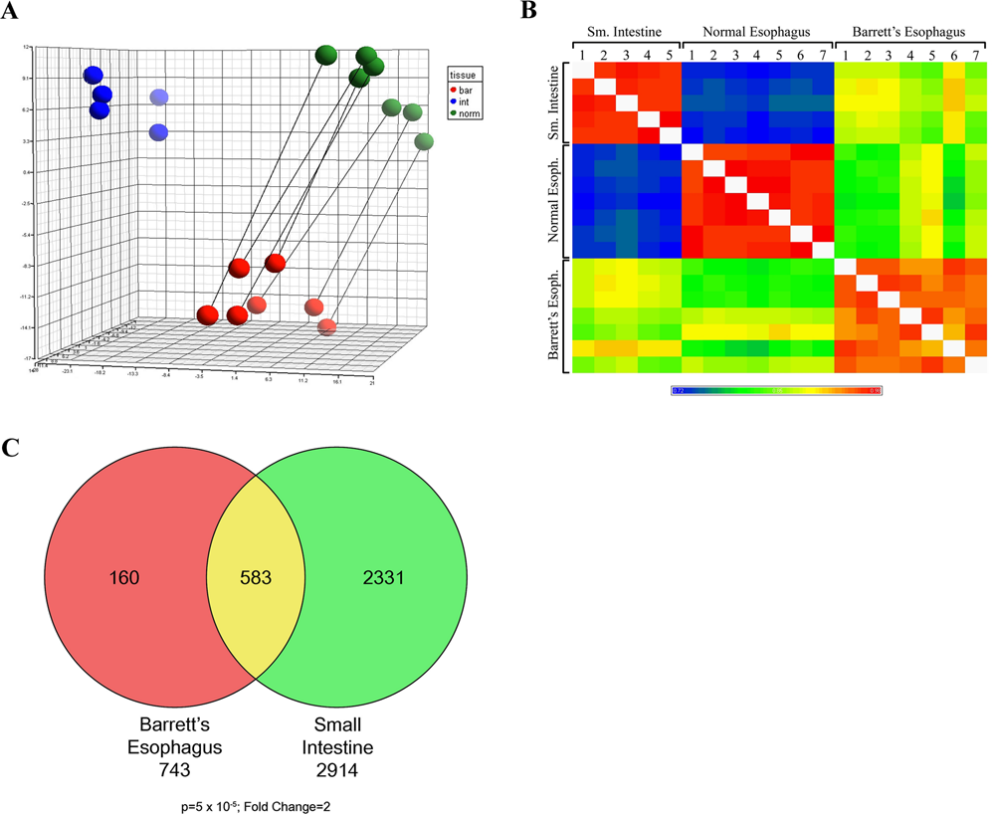
Analysis of microarray data. A. PCA analysis of microarray data generated from 7 matched normal and Barrett's Esophagus and 5 non-related small intestine biopsies. Blue, green and red balls represent small intestine, normal esophagus and Barrett's respectively. B. Pearson correlation analysis of microarray data. C. Venn diagram representing the overlap of genes that are significantly different (Fold change = 2; p value = 5×10^−5^) in Barrett's Esophagus and small intestine relative to normal esophagus.

To begin to identify genes involved in the transdifferentiation process we analyzed Barrett's esophagus (with no dysplasia), normal esophagus and small intestine biopsy samples by Affymetrix microarray. PCA analysis of the array data revealed reproducibility of all samples (Figure 1A). Pearson correlation was performed to analyze similarities between samples and revealed that as a group, Barrett's samples are more heterogeneous than are the two reference groups (Figure 1B). Additionally, Barrett's esophagus is only slightly more similar to small intestine than it is to normal esophagus in terms of the RNA expression profile. This is surprising due to the striking shift in morphology of Barrett's esophagus toward a cell fate very reminiscent to that of the small intestine. The means of the different pairwise comparisons are summarized in Table S1. Same tissue comparisons have a 0.97 correlation coefficient while Barrett's esophagus samples are somewhat less homogenous with a correlation coefficient of 0.95. Comparisons of Barrett's esophagus with normal esophagus and small intestine samples are 0.84 and 0.87, respectively. As a reference value, the comparison of two normal tissues has a correlation coefficient of 0.74. Next, gene lists were generated comparing either Barrett's esophagus or small intestine to normal esophagus expression profiles. A Venn diagram demonstrates the overlap in genes that are significantly altered (fold change = 2; p value<1×10^−5^) (Figure 1C).

**Figure 2:**
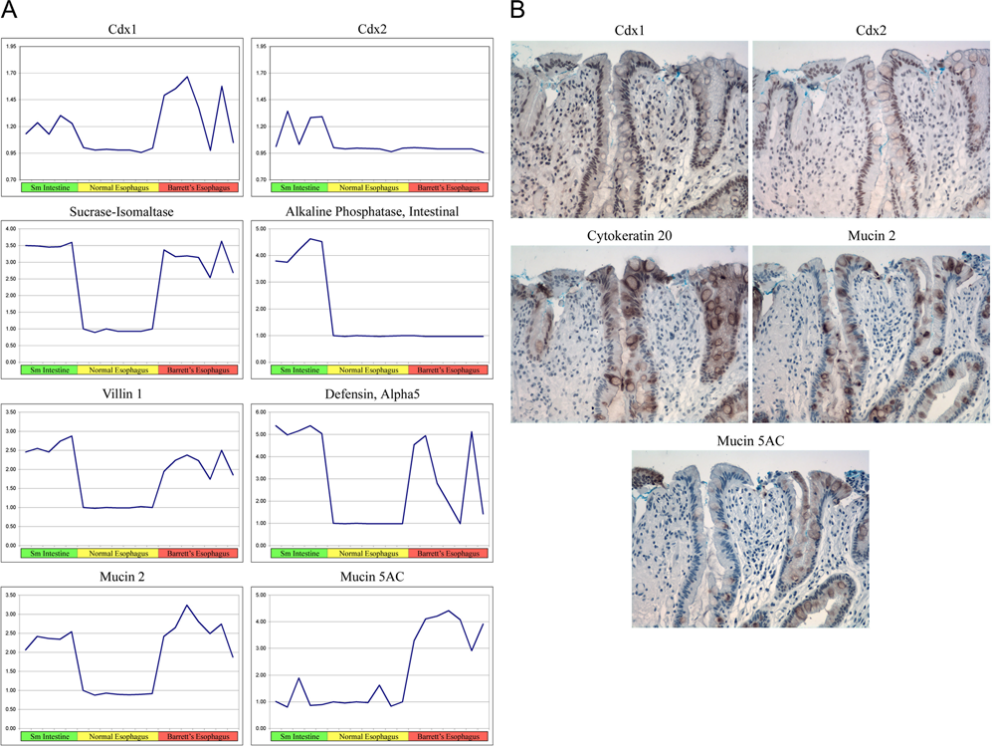
Common Markers of Barrett's Esophagus. A. Expression analysis of 8 common markers of Barrett's Esophagus is represented graphically. Green, yellow, and red bars represent Small intestine, normal esophagus and Barrett's Esophagus respectively. B. Immunohistochemistry of 5 common markers of Barrett's Esophagus on serial sections of an esophageal adenocarcinoma surgical resection sample from a Barrett's Esophagus patient.

Several classic Barrett's esophagus markers have been identified previously. The performance of these markers was analyzed and demonstrates that most of the markers are altered significantly in our data sets (Figure 2A). Interestingly, two classic markers, Cdx2 and Intestinal Alkaline Phosphatase, were not detected in the samples analyzed and suggest that molecular analysis of one endoscopic biopsy sample per patient may not be sufficient to detect all gene profile changes that may have occurred in each patient. To further support this hypothesis of clonal gene expression we analyzed surgical resection samples for Cdx1, Cdx2, Muc2, Muc5AC and CK20 by immunohistochemistry (Figure 2B). Regional expression was detected in these samples for all markers and further supports the notion of clonal gene expression. Alternatively, there may be post-transcriptional or translational regulatory mechanisms that impact upon mosaic gene expression.

An analysis of keratin expression reveals significant changes from those cytokeratins normally expressed in stratified squamous epithelia to those expressed in columnar epithelium (Table 1). Specifically, Keratin 13 is down-regulated 40 fold and Keratins 8 and 18 are upregulated 85 and 11 fold respectively. Additionally, a variety of mucins are expressed in Barrett's esophagus, notably MUC5AC, which is normally expressed in the gastric epithelium (Table 1).

**Table 1:** Keratin and Mucin Affymetrix Data

Squamous Expressed Genes		
	Fold change	p-value
KRT1	−180	7.90E-05
KRT4	−25	2.70E-02
KRT5	−123	6.00E-03
KRT6A	−32	1.70E-02
KRT6B	−84	8.00E-03
KRT10	−2.7	4.40E-04
KRT13	−40	2.00E-02
KRT14	−17	3.30E-02
KRT15	−67	2.00E-03
KRT16	−34	1.00E-03
KRT17	−6.4	1.60E-02
KRT23	−4.3	3.60E-02
KRT24	−22	3.00E-03
**Columnar Expressed Genes**		
	**Fold change**	**p-value**
KRT7	4.6	4.67E-03
KRT8	85	3.31E-08
KRT18	10.9	5.59E-05
KRT19	3.4	1.05E-03
KRT20	369	6.09E-07
TFF1	294	4.50E-05
TFF2	203	1.81E-05
TFF3	508	1.11E-06
MUC2	106	4.58E-05
MUC5AC	289	7.49E-06
MUC13	164	4.96E-08
CD164 - sialomucin	6.8	1.71E-06

Pathway analysis of the gene lists revealed alterations in the expression of several genes involved in the myc pathway, including several myc target genes (Table 2), although c-myc RNA expression was not changed. Both CA2 and ODC1 are upregulated 35 fold in Barrett's epithelium. Of interest are the expression changes of the myc inhibitors, MXD1 (MAD1) and MXI1 (MAD2), which are down regulated 27 and 3.7 fold, respectively. It is possible that c-Myc protein upregulation may be due to MXI1 or MXD1 downregulation. This may lead to enhanced c-myc protein expression. Indeed, c-myc protein has been reported to be increased in Barrett's esophagus [31], [32].

**Table 2:** Affymetrix analysis of the Myc Pathway

	Fold Change	p-value
MXD1 (MAD1)	−27	3.84E-07
MXI1 (MAD2)	−3.7	4.82E-05
MYCN	3.1	2.97E-04
ODC1	36.3	2.06E-05
CA2	34.4	2.38E-05

**Figure 3:**
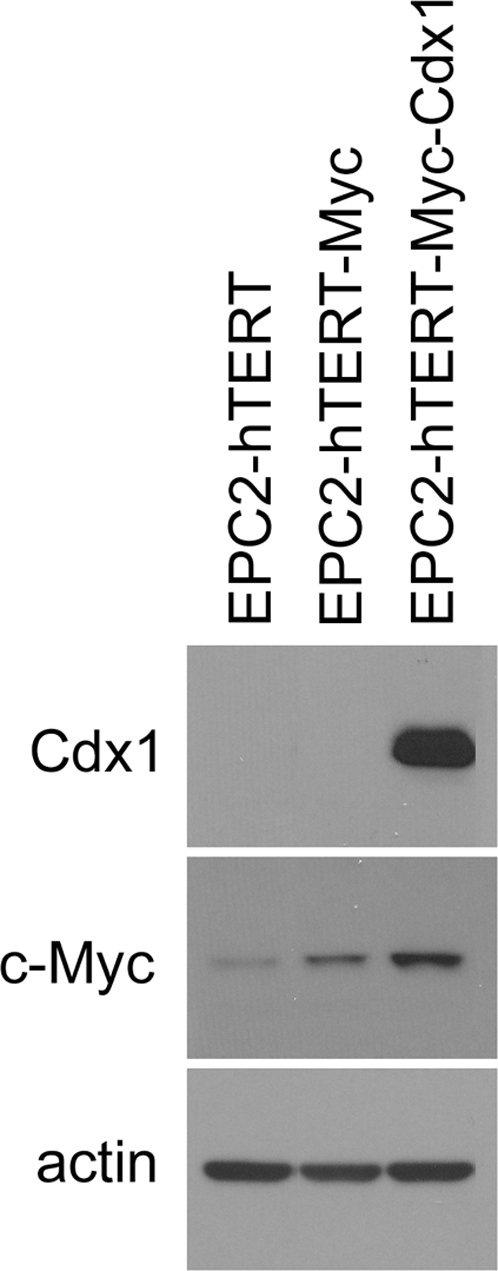
Western analysis of EPC2-hTERT esophageal keratinocytes overexpressing c-myc alone or with Cdx1.

Given that myc activity is involved in the differentiation programs of several cell types, we hypothesized it may play a role in the transdifferentiation process underlying Barrett's esophagus. Likewise, Cdx1 may be functionally related to the development of Barrett's esophagus. To test these hypotheses, we transduced an immortalized esophageal keratinocyte cell line, EPC2-hTERT [33], with c-myc alone or in combination with Cdx1. Western blot analysis confirms overexpression of c-myc and Cdx1 in EPC2-hTERT Myc-Cdx1 cells (Figure 3).

**Figure 4:**
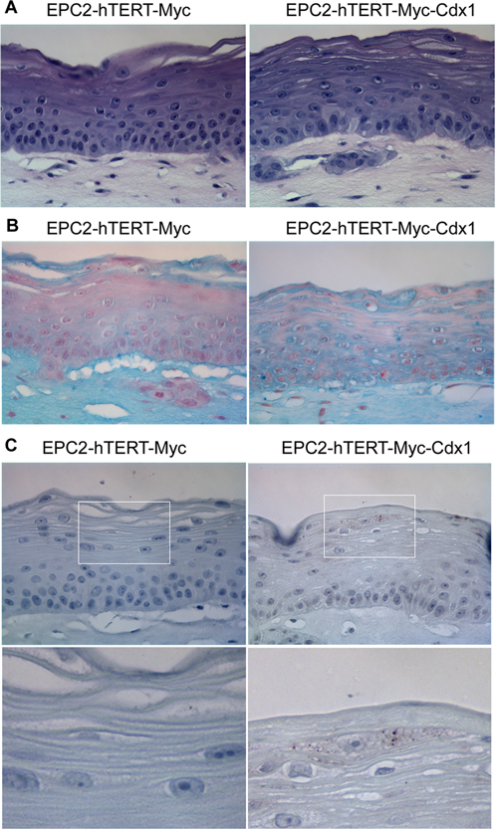
Analysis for mucin expression in EPC2-hTERT-Myc-Cdx1 cells in organotypic culture. A. H&E analysis demonstrates the stratified squamous morphology of keratinocytes in organotypic cultures. B. Alcian blue staining for mucin demonstrates mucin production only in EPC2-hTERT-Myc-Cdx1 cells. C. EPC2-hTERT-Myc-Cdx1 cells are producing Muc5AC as demonstrated by immunohistochemistry for Muc5AC.

In order to model Barrett's Esophagus, EPC2-hTERT Myc cells alone or with Cdx1 were cultured in a 3-dimentional culture system (organotypic culture) previously utilized in our lab to mimic the microenvironment of the esophagus to study esophageal tumorigenesis [34]. This system also recapitulates faithfully the differentiation programs of the stratified epithelium of the esophagus when using non-transformed EPC2-hTERT cells [34]. Analysis of Myc, Myc-Cdx1 cells by histopathology reveals no obvious changes in the stratification of the keratinocytes. However, Alcian blue staining reveals a striking difference in mucin production in these cells but not in parental EPC-hTERT cells (Figure 4A and Figure S1). Specifically, only EPC-hTERT-Myc-Cdx1 expressing cells have a subpopulation of cells that is mucin-producing (Figure 4B). Immunohistochemistry staining of Muc5AC (one of the mucins produced in Barrett's esophagus) reveals its expression in these cells (Figure 4C).

**Figure 5:**
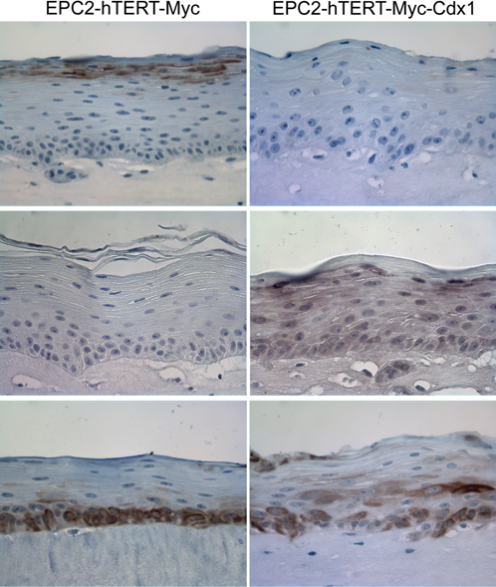
Keratin expression in EPC2-hTERT esophageal keratinocytes overexpressing c-myc alone or with Cdx1. Immunohistochemistry for Keratin 13, 8 and 19 was performed and demonstrates that EPC2-hTERT-Myc-Cdx1 cells have altered keratin expression.

The basal cell layer of esophageal keratinocytes can be identified with Keratins 14 or 19 (Figure 5 and data not shown). Intermediate and upper layers of keratinocytes in organotypic culture can be identified with Keratin 13 staining (Figure 5). Keratin expression analysis also demonstrates an altered gene expression profile specifically in EPC-hTERT-Myc-Cdx1 expressing cells. Keratin 13 is downregulated while Keratin 8 is induced (Figure 5). Keratin 19, expressed in simple columnar epithelium and in the basal layer of stratified squamous esophageal keratinocytes, was also evaluated. In addition to its disrupted expression in basal cells, its expression is expanded into the suprabasal region of Myc-Cdx1 expression cells.

## Discussion

Taken together, these data suggest that c-myc and Cdx1 transcription factors may contribute to in the transdifferentiation process leading to Barrett's Esophagus. The combinatorial expression of these genes leads to one of the two hallmark morphologic features of Barrett's esophagus, namely the production of mucin in a subset of cells (goblet cells in Barrett's esophagus). The other morphologic feature not identified in these cells is the presence of columnar cells. This suggests two non-mutually exclusive hypotheses. One is that other genetic alterations are required for the shift in morphology. The second hypothesis is that the upregulation of these genes precede the changes toward a columnar morphology seen in Barrett's esophagus and still require other genetic alterations. Analysis of stratified squamous epithelium near Barrett's Esophagus provides some evidence for this. Cdx1, but not Cdx2, expression is detected focally in this transitional region (Figure S2). This, in combination with the fact that c-myc is already expressed in esophageal keratinocytes, suggest that c-myc and Cdx1 may cooperate in some of the very earliest initiation stages towards Barrett's Esophagus.

Interestingly, some evidence exists for the cooperation of c-myc with other transcription factors in epithelial transdifferentiation in other tissues [35]–[38]. In the case of gastric intestinal metaplasia, c-myc overexpression is observed similar to that for Barrett's Esophagus. C-myc expression analysis of atrophic gastritis samples with mild and severe intestinal metaplasia reveal c-myc expression in 15% and 47% of samples respectively [38]. In the lung, a squamous metaplastic process occurs before the development of squamous non-small cell lung cancer. Upregulation of c-myc expression in squamous metaplasia is observed with further augmentation as these lesions progress toward squamous cancer [35].

If c-myc is indeed serving as a transcription factor that helps other tissue-specific transcription factors to induce metaplasia, it will be important to understand its role in these processes. This may be achieved through direct or indirect interaction between c-myc and Cdx1 with the induction of target genes. The notion of myc serving as a potentiating transcription factor in a transdifferentiation program is an attractive hypothesis. In addition to the ability of c-myc to bind 10–15% of the promoters in the human genome, it is likely to affect the transcription of even a larger proportion of the genome by its ability to regulate methylation patterns [39]–[42]. This notion of remodeling the genome and altering the differentiation state and cell fate of adult tissues is further supported by c-myc's ability to cooperate in generating induced pleuripotent stem (iPS) cells and an increased number of tumor stem cells when myc is overexpressed [43]–[46].

**Figure 6:**
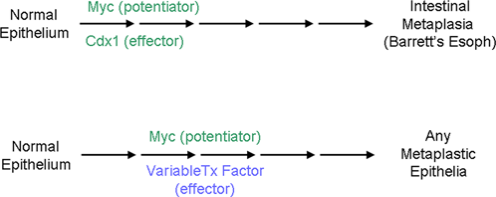
Model of the initial stages of the transdifferentiation towards Barrett's esophagus.

We believe that c-myc and Cdx1 cooperate to induce the initiation of a normal stratified squamous epithelium towards a transdifferentiated state and that Barrett's esophagus requires additional genetic events for the development of a fully mature transdifferentiation process (Figure 6). Several potential transcription factors and signaling pathways are attractive candidates based upon their functional roles in intestinal differentiation; two such pathways are Notch and Hedgehog signaling. Their roles in Barrett's esophagus are under investigation.

## Methods

### Sample collection and Affymetrix analysis

Patients at the Hospital of the University of Pennsylvania undergoing upper endoscopy were enrolled in the study under IRB approval. Four biopsies were obtained each from both regions of endoscopically appearing Barrett's esophagus and normal esophagus. Each set of biopsies was placed in RNA Later (Ambion) for processing. Each biopsy was divided with a section processed for RNA isolation and one for histology. RNA was isolated by first homogenizing the tissue with a Brinkmann homogenizer, passing it through a QIAshredder (Qiagen,) column and isolating the RNA by RNeasy miniprep kit (Qiagen). Sample collection and RNA extraction were performed as above for normal small intestine biopsies. The formalin-fixed tissue samples were processed, embedded, sectioned and stained with hemotoxylin and eosin, and evaluated in a blinded fashion for pathologic diagnosis. Seven paired patient samples were selected for microarray analysis based on the quality of RNA and presence of intestinal metaplasia without gastric mucosa or dysplasia. cRNA was prepped according to Affymetrix recommended protocols and run on U133A version 1 chips. Since samples were not all prepped simultaneously, they were grouped into 3 batches of experimental samples for analysis in the 3-way mixed model ANOVA (see below). Microarray data (accession # GSE13083) was deposited with Gene Expression Omnibus at the NCBI (http://www.ncbi.nlm.nih.gov/geo).

### Bioinformatics analysis

These 19 Cel files were processed using Stratagene's Array Lite 3.4 software using GC-RMA algorithm to calculate probeset intensity values and to calculate Absent/Present/Marginal flags. These intensity values were log_2_ transformed and then, GC-RMA values were imported into Partek Genomics Suite 6.3 (beta) retaining only those probesets flagged as present in at least 3 of 19 samples. A 3-way mixed model ANOVA was performed and included a pairwise contrast that was patient matched between normal esophagus and Barrett's Esophagus. In a second analysis, a 1-way ANOVA was done and simultaneously performed a pairwise contrast between normal small intestine and normal esophagus. For each analysis p-values and fold change for probeset were calculated. Principal component analysis (PCA) and Pearson correlation analyses were executed using all probesets that passed the 3 out of 19 present call filtering with no other limitations places on each data set.

### Cell Culture

Primary human esophageal keratinocytes, designated as EPC2, were established as described previously[33]. Cells were maintained at 37°C and 5% CO_2_ using keratinocyte-SFM medium (KSFM; Invitrogen) supplemented with 40 µg/mL bovine pituitary extract (Invitrogen), 1.0 ng/mL EGF (Invitrogen), 100 U/mL penicillin, and 100 µg/mL streptomycin (Invitrogen). Stable transduction of primary esophageal cells with retroviral vectors was described previously [33], [47], [48]. Vectors used were LXSN, LMycSN, MIGR-Cdx1 and MIGR-Cdx2. Cells were passaged 48 h after infection and selected with 300 µg/mL G418 (Invitrogen) for a period of 7 days. Following selection, cells were FACS sorted and GFP-positive cells were obtained.

### Organotypic Culture

Organotypic culture was performed as previously described [34]. Briefly, a collagen/Matrigel matrix, containing 76.7% bovine tendon acid-extracted collagen (Organogenesis), Matrigel Matrix (BD Bioscience), 1× minimal essential medium with Earle's salts (BioWhittaker), 1.68 mM L-glutamine (Cellgro), 10% fetal bovine serum (Hyclone), 0.15% sodium bicarbonate (BioWhittaker) was mixed with 7.5×10^4^ human fetal esophageal fibroblasts. Following 7 days, 5×10^5^ human esophageal keratinocytes were seeded on top of the matrices. Cultures were fed with Epidermalization I medium for 2 days, which is a 3∶1 mixture DMEM (JRH Biosciences)/Ham's F-12 (Invitrogen) supplemented with 4 mM L-glutamine, 0.5 µg/mL hydrocortisone, 0.1 mM O-phosphorylethanolamine, 20 pM triiodothyronine, 0.18 mM adenine, 1.88 mM CaCl2, 4 pM progesterone (Sigma); 10 µg/mL insulin, 10 µg/mL transferrin, 5 mM ethanolamine, 10 ng/mL selenium (ITES) (BioWhittaker), and 0.1% chelated newborn calf serum (Hyclone). For the next 2 days, cultures were fed with Epidermalization II medium, which is identical to Epidermalization I medium except that it contains 0.1% unchelated newborn calf serum. Then, cultures were raised to an air–liquid interface and cultured for 4 days in Epidermalization III medium, which contains the same growth supplements as Epidermalization I and II except that no progesterone is added and 2% newborn calf serum is used. Cultures were then harvested by fixing in neutral buffered formalin and later were paraffin-embedded.

### Western Analysis

For Western blot analysis, cells were lysed in lysis buffer (10 mM Tris·HCl at pH 7.4, 150 mM NaCl, 1% Nonidet P-40, 0.1% sodium deoxycholate, 0.1% SDS, 1 mM EDTA, 2 mM sodium orthovanadate, protease inhibitor tablet (Roche Molecular Biochemicals). Protein concentration was determined by the Bio-Rad protein assay. Fifteen micrograms of protein were run on a 4%–12% SDS-PAGE gel (Invitrogen) and transferred to a PVDF membrane (Immobilon-P; Millipore). Membranes were blocked in 5% nonfat milk (Bio-Rad) in PBS with 0.05% Tween 20 (PBS-T) for 1 hr at room temperature, then probed with primary antibody diluted in 5% milk in PBS-T overnight at 4°C, washed with PBS-T, incubated with HRP-conjugated secondary antibodies (GE Biotech; 1∶5000 in PBS-T) for 1 h at room temperature, and washed in PBS-T. The signal was visualized using ECL Plus (Amersham Pharmacia Biotech) and exposed to Blue Lite Autorad film (ISC-BioExpress). Primary antibodies used were: Cdx1 (ab2400, Abcam), c-myc (NCL-cMYC, Lab Visions), beta-actin (A5316, Sigma).

### Immunohistochemistry

Immunohistochemistry was performed with the Vecta Elite kit (Vector Laboratories) following the manufacturer's protocol. Briefly, paraffin sections were dewaxed with xylene and microwaved for 10 min in the presence of 10 mM citric acid buffer. Endogenous peroxidases were quenched using hydrogen peroxide before sections were blocked in avidin D-blocking reagent and biotin-blocking reagent. Sections were incubated with primary antibody overnight at 4°C and secondary antibody for 30 min at 37°C, and then signal was developed using the DAB substrate kit for peroxidase (Vector Laboratories).Antibodies used were: Cdx1(ab2400, Abcam), Cdx2 (MU392A-UC, Biogenex), Muc2 (VP-M656, Vector Labs), Muc5AC (VP-M657, Vector Labs), Keratin 20 (M7019, DakoCytomation), c-myc (NCL-cMYC, Lab Visions), Keratin 13 (NCL-CK13, Lab Visions), Keratin 19 (TROMA-III, Univ of Iowa Developmental Studies Hybridoma Bank), Keratin 8 (RDI-PRO61038, Research Diagnostics).

### Real-time PCR

RNA was isolated from cell lines using the RNeasy mini kit (Qiagen) according to manufacturer's instructions. cDNA synthesis and amplification were performed using the WT-Ovation RNA amplification System (NuGen Technologies) according to manufacturer's instructions. Real-time PCR was performed and analyzed using ABI PRISM 7000 sequence detection system software (PE Applied Biosystems) using the Power SYBR Green PCR Master Mix (PE Applied Biosystems) for Actin according to the manufacturer's instructions. Taqman assays were used with the Taqman Universal PCR Master Mix (PE Applied Biosystems) according to manufacturer's instructions. The following Taqman assays (PE Applied Biosystems) used were: TFF1 (Hs00170216_m1), TFF2 (Hs00193719_m1), TFF3 (Hs00173625_m1), Muc2 (Hs00159374_m1), Muc5AC (Hs01365601_m1), Muc13 (Hs00217230_m1), CD164 (Hs00174789_m1), ODC1 (Hs00159739_m1), CA2 (Hs00163869_m1). Actin primers were: Forward: CCT GGC ACC CAG GAC AAT, Reverse: GCC GAT CCA CAC GGA GTA CT.

## Supporting Information

Figure S1 Alcian blue staining of EPC2-hTERT cells. Mucin staining of the parental cell line EPC2-hTERT is negative.
(9.38 MB TIF)

Figure S2 Immunohistochemical analysis of stratified squamous epithelium adjacent to Barrett's esophagus. Cdx1, Cdx2 and c-myc staining of the stratified squamous epithelium demonstrates focal staining of Cdx1 which co-localizes with intense nuclear c-myc staining. Cdx2 is not expressed in these regions. Circles represent the same areas from serial sections.
(9.82 MB TIF)

Table S1 (0.01 MB DOC)
