# Supplementary figures and images for: Cdx1 and c-Myc Foster the Initiation of Transdifferentiation of the Normal Esophageal Squamous Epithelium toward Barrett's Esophagus
Source: PLoS One. 2008 Oct 27;3(10):e3534. doi: 10.1371/journal.pone.0003534 (PMC2568822; doi:10.1371/journal.pone.0003534)

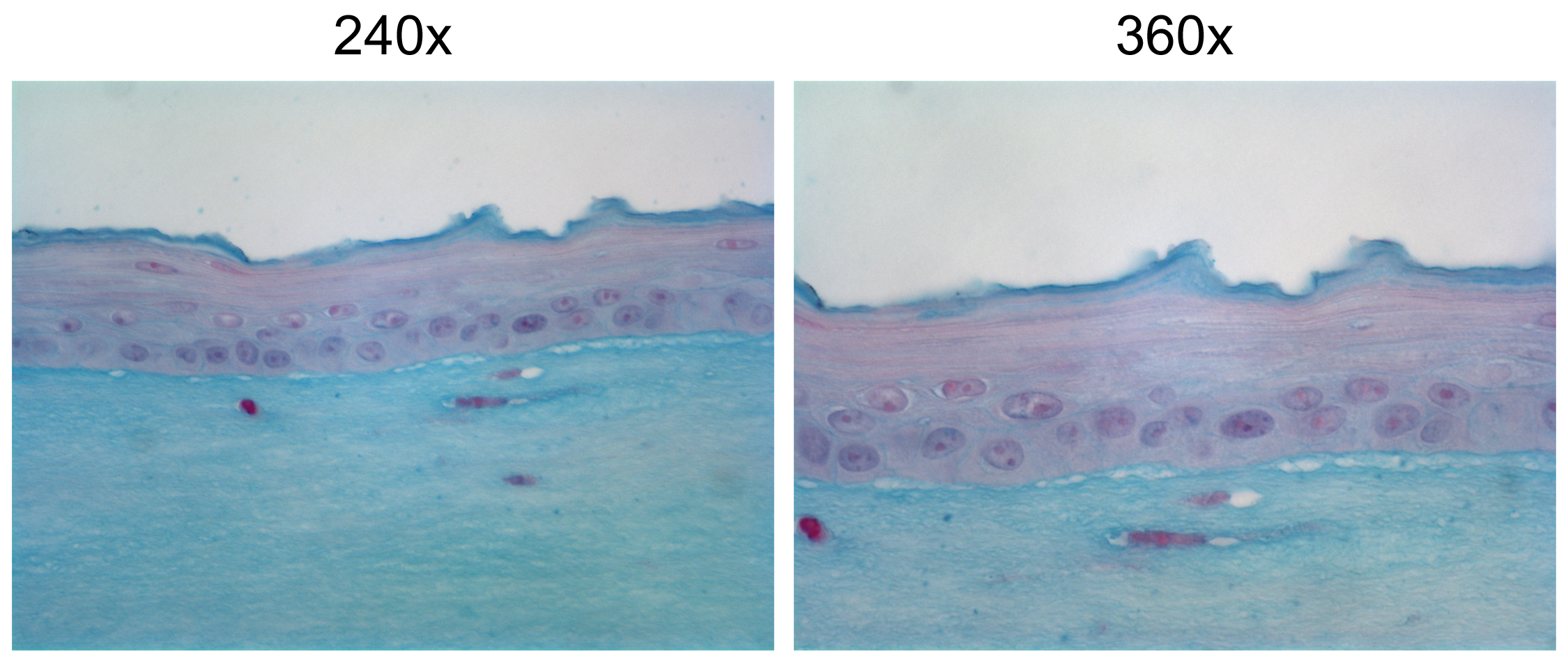

Supplement: Figure S1 — Alcian blue staining of EPC2-hTERT cells. Mucin staining of the parental cell line EPC2-hTERT is negative. (9.38 MB TIF) [file pone.0003534.s001.tif]

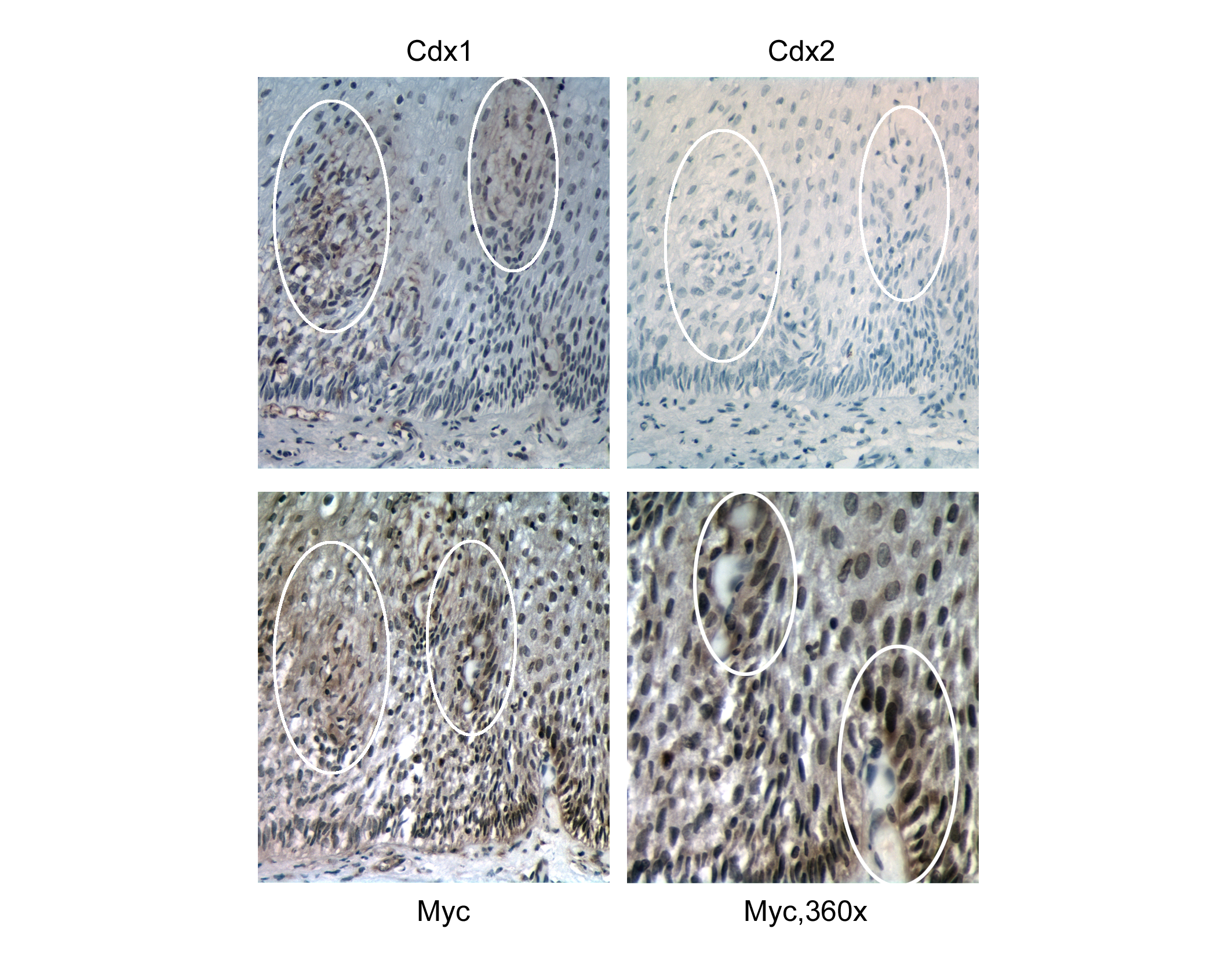

Supplement: Figure S2 — Immunohistochemical analysis of stratified squamous epithelium adjacent to Barrett's esophagus. Cdx1, Cdx2 and c-myc staining of the stratified squamous epithelium demonstrates focal staining of Cdx1 which co-localizes with intense nuclear c-myc staining. Cdx2 is not expressed in these regions. Circles represent the same areas from serial sections. (9.82 MB TIF) [file pone.0003534.s002.tif]
