# Supplementary material for: Cdx1 and c-Myc Foster the Initiation of Transdifferentiation of the Normal Esophageal Squamous Epithelium toward Barrett's Esophagus
Source: PLoS One. 2008 Oct 27;3(10):e3534. doi: 10.1371/journal.pone.0003534 (PMC2568822; doi:10.1371/journal.pone.0003534)
Supplement: Table S1 — (0.01 MB DOC) [file pone.0003534.s003.doc]

**Supplemental Table 1.** Mean values of the pairwise correlations

| **Comparison** | **Mean Correlation** |
| --- | --- |
| NE vs NE | 0.97 |
| Int vs Int | 0.97 |
| BE vs BE | 0.95 |
| BE vs NE | 0.84 |
| BE vs Int | 0.87 |
| NE vs Int | 0.74 |
